# Mitigate the variation of energy band gap with electric field induced by quantum confinement Stark effect via a gradient quantum system for frequency-stable laser diodes

**DOI:** 10.1515/nanoph-2025-0380

**Published:** 2025-12-09

**Authors:** Yuhong Wang, Yiwei Zhang, Zihan Jiang, Jian Wu, Chunqing Gao

**Affiliations:** School of Optics and Photonics, 47833 Beijing Institute of Technology, Beijing 100081, China; Key Laboratory of Information Photonics Technology, Ministry of Industry and Information Technology, Beijing 100081, China; School of Physics, Beihang University, Beijing 102206, China

**Keywords:** frequency-stable laser diodes, quantum-confined Stark effect, energy band gap, indium-segregation effect

## Abstract

Most light-emitting devices based on quantum-confined structures are commonly utilized as electrically injected devices. However, the electric-field-dependent energy band gap induced by the quantum confinement Stark effect (QCSE) usually hinders the realization of frequency-stable laser devices. This is because the change in the energy band gap, which also means the corresponding change in the photon energy, will result in an electric-field-dependent frequency. Here, we propose a novel approach to mitigate this electric-field-dependent variation in the energy band gap by employing a gradient quantum system. In this system, the energy band edges are inclined due to the action of the indium (In)-segregation effect. This special design can effectively weaken the changes in the band profile associated with the electric field effect and counteract the electric-field-dependent band gap variations within the active region to a certain extent. Experimental studies indicate that the energy band gap of this gradient quantum system remains almost unchanged (<18.9 μeV cm^2^/A) even under a relatively strong applied electric field. Meanwhile, compared with the traditional GaAs quantum well, the efficiency improvement in the band gap stability of our nanowire–well gradient system is 64.1 % and 70.6 % for the TE and TM polarization modes, respectively, which suggests that our proposed gradient quantum structure can significantly mitigate the electric-field-induced change in the energy band gap. This achievement is of great significance for advancing the development of high-performance frequency-stable laser devices in some advanced fields, such as quantum sensing systems and optical communications.

## 1 Introduction

It is well known that most of the light-emitting devices based on quantum-confined structures work under the application of an additional electric field, as the electrically injected devices [1]. However, in these devices, due to the presence of the additional vertical electric fields, the conduction and valence band edges in semiconductor active materials are inclined in the same direction, reducing the energy band gap, known as the quantum confinement Stark effect (QCSE) [2], [3], [4], [5], [6], [7], [8], [9]. The electric-field-dependent energy band gap caused by the QCSE is unfavorable for the realization of frequency-stable laser devices. This is because the variation in the energy band gap in the active material with an electric field means the corresponding change in photon energy. And the variation in photon energy will result in an injection-dependent frequency. Therefore, the QCSE limits the achievement of frequency-stable laser devices. However, ultra-stable laser frequencies have served as the prerequisite behind some advanced fields, such as quantum sensing systems, optical communications, optical detection, and precise experiments [10], [11], [12], [13].

In addition, since the strained II–VI and III–V compounds that served as the active materials in the optoelectronic devices can usually cover a more extensive operating wavelength range [14], [15], the strained optoelectronic devices have received widespread application in many areas [15], [16], [17], [18], [19]. However, a similar QCSE phenomenon also occurs in these strained material systems owing to the presence of strain-driven built-in piezoelectric polarization electric fields in these material systems [3], [4], [5].

Given the ubiquity of the QCSE in quantum-confined devices and the negative impacts this effect brings, which are not limited to the electric-field-dependent energy band gap, but also include inefficient radiative recombination, many efforts have been made to circumvent this effect [3], [20], [21]. Previous efforts to mitigate the influence of the QCSE have mainly focused on nitride (N)-based materials, as the spontaneous polarization fields of the polar (In)GaN materials themselves, the strain-driven piezoelectric polarization fields, and the applied external electric fields in these material systems are responsible for the QCSE [2], [3]. Based on these strained N-based materials, several specific structure designs have been proposed to minimize the QCSE including the nonpolar/semi-polar structures that aim at minimizing the spontaneous polarization fields [22], [23], [24], the lattice-matched AlInGaN/AlInGaN multiple quantum wells and the strain-compensated InGaN/AlGaN quantum well designed to reduce strain-related effect impact [25], [26], as well as several quantum structures with various shapes designed by energy band engineering to comprehensively attenuate the QCSE influence driven by the built-in and external applied electric fields, such as staggered well [3], [27], triangular quantum well [28], dip-shape well [29], and trapezoidal well [21]. In addition, the employment of thinner quantum wells has previously been suggested to attenuate the QCSE, but it is generally inefficient to capture the carriers into the thin quantum wells, leading to an overall lower internal quantum efficiency [22], and thus, thinning the quantum wells to mitigate the QCSE is not recommended in high-performance optoelectronic devices. Consequently, considering the complexity in regulating the growth conditions multiple times during the preparation of these specially designed structures, the difficulty of accurately controlling the material compositions, as well as the practicability in practical applications, a kind of novel quantum structure or material system that can be obtained using simple fabrication methods and growth conditions is urgently needed in reducing the QCSE impacts to obtain mass-produced high-performance devices.

Given the above considerations, we propose and recommend a gradient quantum structure with the opposite tilt-direction for conduction and valence band edges to mitigate the electric-field-dependent variation in energy band gap induced by the QCSE. This new material structure is designed and fabricated in our previous research work and exhibits many interesting properties, such as super-gain spectra, polarization insensitivity, temperature-insensitive energy band gap, and so on [30], [31], [32], [33]. In the latest research presented here, the mitigation of the impact of vertical electric field on energy band gap variation induced by the QCSE in this special structure is observed by modulating the energy band structure utilizing the In-segregation effect. In-segregation is a strain-driven effect, and its occurrence is usually limited in material growth as a growth defect due to its altered In-contents. Here, however, the In-segregation effect is effectively utilized. The band edge tilt driven by the In-segregation effect effectively counteracts the band edge tilt caused by the electric field for the valence band in this system. The modified energy band profile in the gradient system makes the energy band gap of the active material almost invariant with the change in the electric field. The relevant analysis and discussion of the mitigation of the electric-field-dependent energy band gap in this gradient quantum system utilizing the In-segregation effect are given in the following sections.

## 2 Material structure and characterization

### 2.1 Material structure

**Figure 1:**
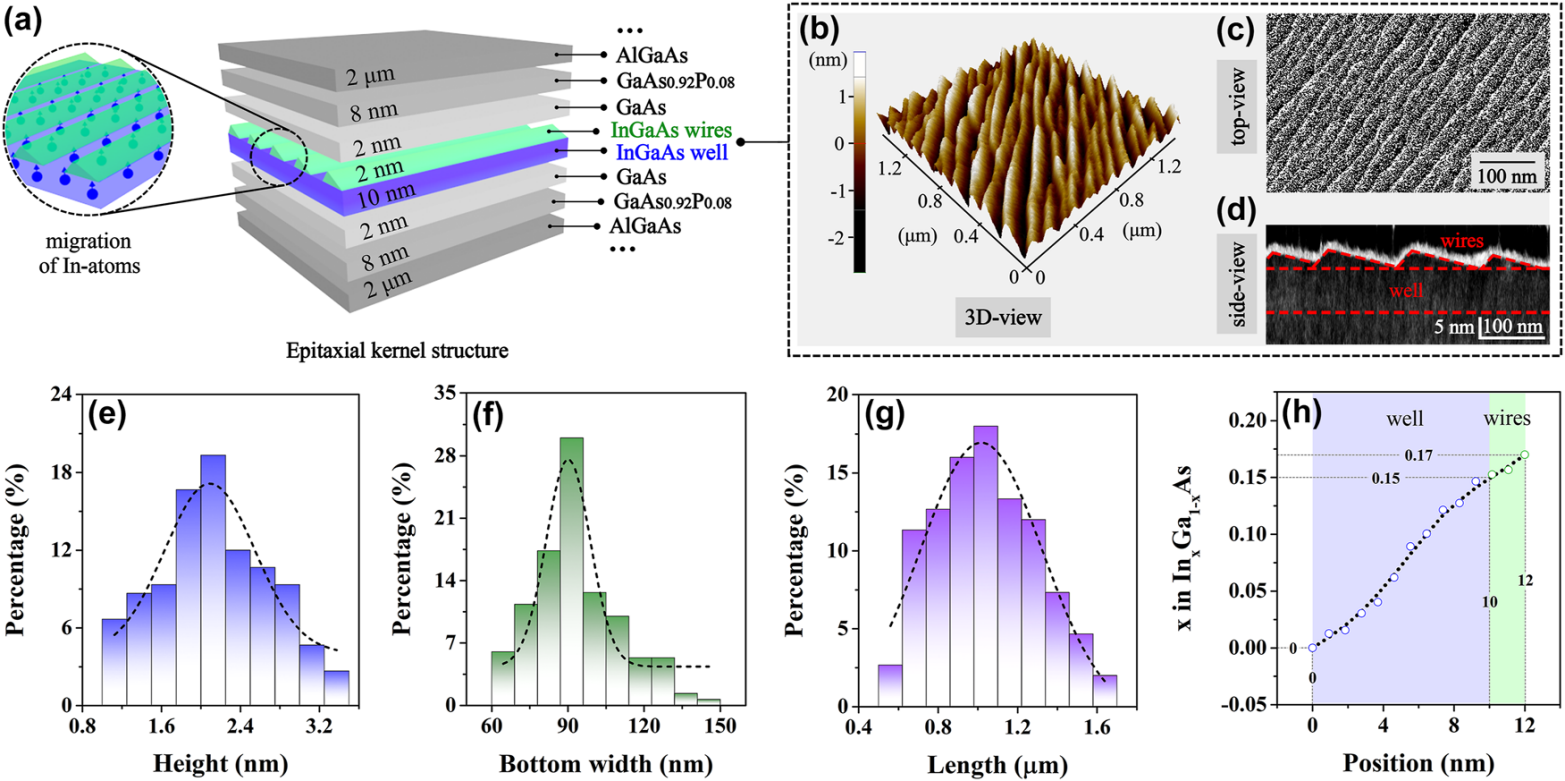
Material structure and characterization. (a) Schematic diagram of the structural characteristics of the fabricated gradient quantum structure. (b) The 3D-view, (c) top-view, and (d) side-view of on-well nanowires based on AFM, SEM, and X-TEM characterizations, respectively. Histograms of the (e) height (blue pillars), (f) bottom width (green pillars), and (g) length (purple pillars) distributions and corresponding Gaussian fitting curves (black dashed lines) of InGaAs nanowires by measuring 150 wires at random. (h) Profile of In-content in the nanowire–well structure obtained from the XPS measurement.

Previous research on the mitigation of the QCSE has focused mainly on N-based materials, ignoring the fact that other strained non-(In)GaN materials and the active materials in electrically injected devices are also troubled by the QCSE due to the strain-driven built-in piezoelectric polarization electric fields and externally applied electric fields. It is well known that high-power InGaAs-based lasers are the most representative frequency-stable laser sources. They are widely used for atomic clocks in quantum sensing systems, laser diodes in optical communications, as pumping laser sources for erbium-doped fiber amplifiers and lasers, etc. [10], [34], [35]. Ultra-stable laser frequencies are generally indispensable for these systems. To mitigate the electric-field-dependent frequency drifts induced by the QCSE for InGaAs material systems is particularly important. Therefore, the InGaAs/GaAs/GaAsP quantum structure is chosen as the vehicle in our study. Based on the uniform multiatomic steps that naturally formed on initially deposited 200 nm-thick GaAs surface at a slightly misoriented GaAs (001) substrate [36], [37], [38], the InGaAs nanowire–well structure is formed. The formation of this hybrid structure is completely self-assembled at a step flow growth mode, as well as the 2 nm-high nanowires (one-dimension) and 10 nm-thick well (two-dimension) are combined together through a special dimension-transition interface. In addition, a given In-content of 0.17 is applied to the initial InGaAs material. Since the InGaAs layer is inserted between the GaAs strain-buffer layers, it will experience compressive stress induced by the lattice mismatch, resulting in the occurrence of the strain-driven In-segregation effect in this InGaAs active layer under given experimental conditions. Therefore, a certain amount of In-atoms will migrate from the bottom to the top of the InGaAs layer along the material growth direction [39], [40], [41], which will cause the monotonically changed In-content in both well and wires. The diagram of the In-atoms migration in the nanowire–well structure is displayed in the left schematic representation of Figure 1(a).

Additionally, the schematic illustration of the epitaxial kernel structure is illustrated in the right diagram of Figure 1(a), in which the InGaAs nanowire–well structure is sandwiched by two 2 nm-thick GaAs strain-buffer layers. The insertion of strain-buffer layers can avoid the capture of carriers by dislocations (nonradiative recombination centers) caused by the large lattice mismatches between the active layers and the barriers, affecting the radiative recombination in the active region. Beyond the strain-buffer layers are 8 nm-thick GaAs_0.92_P_0.08_ barriers. Furthermore, the InGaAs/GaAs/GaAsP system is surrounded by 2 μm-thick AlGaAs waveguide layers.

### 2.2 Sample fabrication

This nanowire–well sample is fabricated based on the preparation method described in our previous works by a metal–organic chemical vapor deposition (MOCVD, aix200/4) system [30], [31], [32], [33]. Trimethylindium (TMIn), trimethylgallium (TMGa), arsine (AsH_3_), phosphine (pH_3_), and trimethylaluminum (TMAl) are selected as source materials. The pressure and total flow in the reaction chamber are set to 100 mbar and ∼13 slm, respectively. Moreover, the material growth rate is fixed at ∼1 μm/h, and the V/III ratio is controlled at about 50. The growth temperature is ∼650 °C.

### 2.3 Structural characterizations

The nanowire–well structure is characterized utilizing a special sample with the uncovered InGaAs surface, which is fabricated under the same growth conditions by terminating its epitaxial growth process just after obtaining the complete nanowire–well layer. A three-dimensional (3D) extraction of the nanowires measured in atomic force microscopy (AFM, XE100) is displayed in Figure 1(b), from which the densely and horizontally aligned InGaAs nanowires are demonstrated. To further confirm the surface morphology of the wires, the scanning electron microscope (SEM, Zeiss gemini300) analysis is carried out. A representative top-view image of the nanowires from SEM measurement is shown in Figure 1(c), where the orderly arrangement of wires can be seen. Moreover, to reveal the morphological characteristics in the side-view of the wires, the cross-sectional transmission electron microscopy (X-TEM, JEM-2100F) technique is applied here. The triangle-like nanowire cross sections with a large top-angle and a very small base-angle are presented, as exhibited in the X-TEM image of Figure 1(d), where the nanowire–well structure is outlined with red dashed lines. It should also be mentioned here that the white contour line along the nanowires is formed by the focused-ion-beam (FIB) treatment during the fabrication process of the sample cross section.

The statistical average height of wires is 2.0 nm, with a bottom width and length of 90.0 nm and 1.0 μm, respectively, which are extracted by randomly measuring 150 wires in AFM images and fitting the corresponding statistical average size with a Gaussian distribution (refer to Figure 1(e)–(g)). Furthermore, since the In-content (*x* value) in In_*x*_Ga_1−*x*_As material can most effectively influence the energy band gap [42], the investigation of the In-content distribution in this nanowire–well structure is performed utilizing the X-ray photoelectron spectroscopy (XPS, PHI Quantera II) technology. According to the measurement result, the In-content varies approximately from *x* = 0 to *x* = 0.15 in the well and from *x* = 0.15 to *x* = 0.17 in the nanowires along the material growth direction, as is visible in Figure 1(h), which is consistent with the driving impact of the In-segregation effect. Therefore, the quantum system with gradient changes in the In-content has been achieved in our experiment, which will lead to corresponding changes in the energy band structure. The obtained structural characterizations presented here are found to be consistent with the structural information of the nanowire–well sample we prepared previously under the same fabrication conditions [30], [31], [32], [33].

### 2.4 Structural division

**Figure 2:**
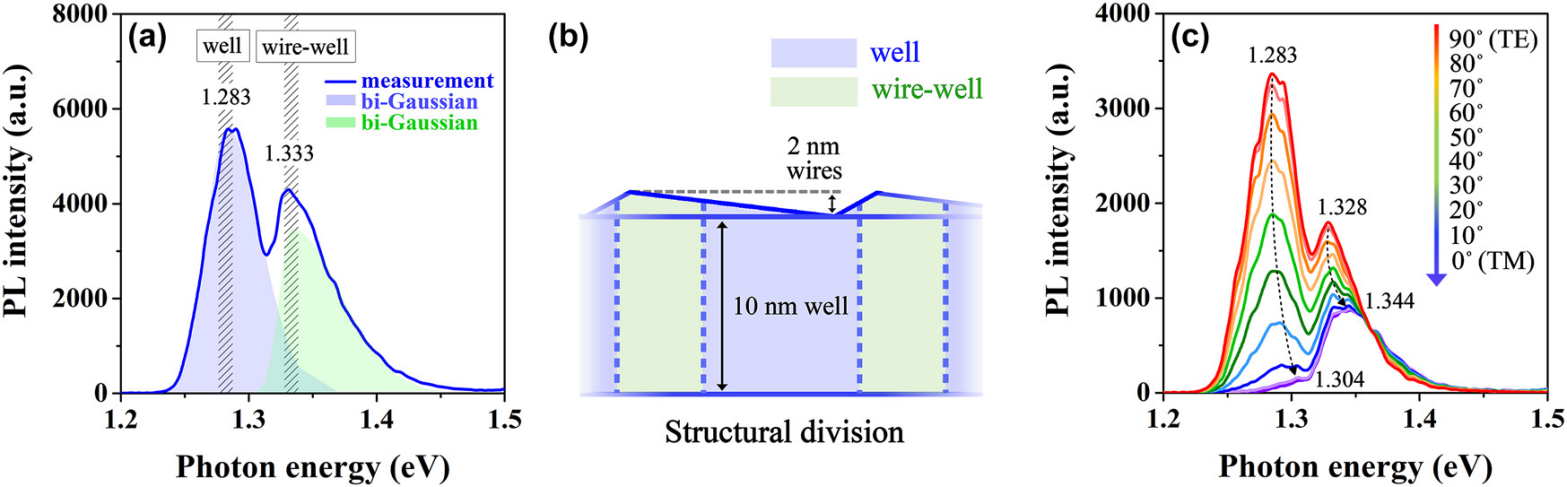
Structural division. (a) PL spectrum analysis of the nanowire–well structure with the experimental data and the bi-Gaussian fittings. (b) Schematic diagram of the structural division in the nanowire–well structure. (c) The changes in PL-peak characteristics with varying polarization from TE to TM modes in the nanowire–well structure.

To investigate the fundamental optical properties of the nanowire–well samples, the photoluminescence (PL) spectrum under 197 mW power injection is conducted at room temperature and depicted in Figure 2(a), in which a pump beam from an 808 nm pulsed laser (LWIRL808-40W-F) is utilized to pump the samples from the top optically. Considering the anisotropic properties of one-dimensional nanowires, all of the PL spectra are measured along the direction parallel to the nanowires to collect the strongest light signals for a nanowire–well edge-emitting chip [38], [43]. A very obvious feature presented in the PL spectrum of Figure 2(a) is the presence of two luminescence peaks, involving energy band gaps around 1.283 eV and 1.333 eV, respectively. Given that the cross section of the nanowires presents an obtuse-triangle-like profile with a large top angle and a very small base angle, as exhibited in Figure 1(d), it is evident that the effective region of the nanowires does not completely cover the well surface, but rather presents a horizontal periodic distribution in space. Consequently, the appearance of two luminescence peaks is attributed to the contributions of the pure-well part without the nanowire coverage (marked as “well”) and the nanowire–well part with nanowire participation (marked as “wire–well”).

The schematic diagram of the structural division is displayed in Figure 2(b). Owing to the confinement of two dimensions, a larger energy band gap will form in the nanowires, compared to the well with the same material and composition but only confined by one dimension [44]. Thus, when the nanowires and the well are directly bound together, their unified energy band gap will be larger than the band gap of a pure well without a nanowire impact. As a result, the peak located at ∼1.333 eV corresponds to the nanowire–well part, and the peak near 1.283 eV is the characteristic PL peak for the pure-well part, as indicated by “wire–well” and “well” in Figure 2(a). The bi-Gaussian curve fittings for extracting the respective luminescence peaks of these two parts are also conducted and indicated in Figure 2(a), in which the luminescence peak of the nanowire–well part is represented by a green shadow, while the luminescence peak of the pure-well part corresponds to the blue shadow position. Here, the PL emission intensity from the nanowire–well part is significantly weaker than that from the pure-well part, as observed in Figure 2(a). This intensity difference between the two luminescence peaks indicates the different proportions of these two parts in the entire active structure.

In addition, since the InGaAs well layer is inserted between the GaAs strain-buffer layers, it will experience compressive stress induced by the lattice mismatch. However, owing to the action of the wires, the tensile stress is introduced into the compressively strained quantum well via the well–wire interface, and the tensile stress counteracts part of the compressive stress in this well, so overall, the influence of the stress impact in the nanowire–well part is very weak. The related analyses have been illustrated in detail in our previous work [32]. The strain-compensated nanowire–well part designed here is aimed at weakening the influence and interference of the strain-driven built-in piezoelectric polarization electric field on the QCSE. Therefore, a more accurate mechanism of weakening the QCSE in this gradient quantum system can be considered and analyzed.

Furthermore, to confirm that the secondary peak at 1.333 eV appears in the PL spectra on account of the presence of the nanowire–well part, rather than the influence of the second energy level in the pure-well part, we measure the polarized PL spectra of this nanowire–well structure as presented in Figure 2(c). Here, the obtainment of transverse electric (TE)- and transverse magnetic (TM)-polarized spectra via rotating the polarizer from 90° to 0°, in which we define the TE polarization as the 90° polarizer angle, i.e., it takes the direction parallel to the sample surface and the TM polarization as the 0° polarizer angle, i.e., it is normal to the sample surface. The two luminescence-peak phenomena still exist when switching the polarization from TE to TM modes, and the main peak in TE polarization shifted from position ∼1.283 eV to position ∼1.304 eV in TM polarization, while the secondary peak shifted from position ∼1.328 eV to position ∼1.344 eV. More importantly, the peak located at ∼1.344 eV in TM polarization has a stronger peak intensity than the peak at ∼1.304 eV. Therefore, the peak at ∼1.328 eV in TE mode and the peak at ∼1.344 eV in TM mode are the contributions of the nanowire–well part rather than the impact of the second energy level of the pure-well part.

## 3 Results and discussions

### 3.1 Mitigated electric-field-dependent variation in energy band gap of the nanowire–well structure

To experimentally clarify the inhibitory effect of this nanowire–well gradient system on the electric-field-dependent variation in energy band gap, two different pumping methods, electric injection and optical injection, were used to test the optical properties of these nanowire–well samples. Here, the external vertical electric field in the nanowire–well electric injection sample is applied to induce an additional QCSE in this nanowire–well structure, whereas the additional QCSE is not present in the optical injection sample. In addition, since the change in energy band gap with injection also corresponds to a change in photon energy, it will result in a change in luminescence peak position. Therefore, by comparing the luminescence peak positions of PL spectra and electroluminescence (EL) spectra, the mitigation degree of electric-field-dependent variation in the energy band gap induced by the QCSE in this structure can be judged.

**Figure 3:**
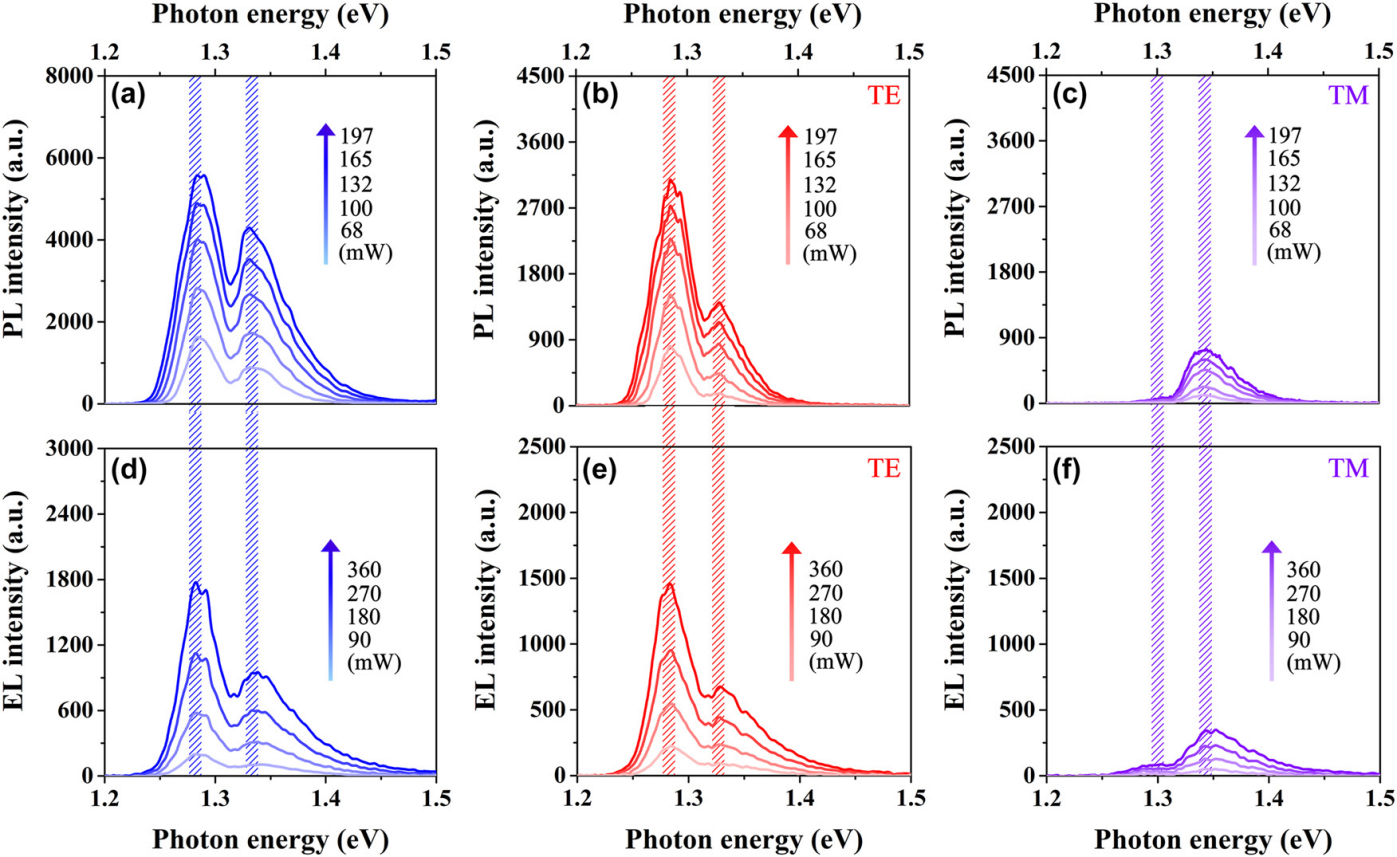
Luminescence peak characterizations of the PL and the EL spectra at similar injection. Injection power-dependent (a) unpolarized, (b) TE-polarized, and (c) TM-polarized PL spectra of the nanowire–well structure from 68 mW to 197 mW at room temperature. Injection power-dependent (d) unpolarized, (e) TE-polarized, and (f) TM-polarized EL spectra of the nanowire–well structure from 90 mW to 360 mW at room temperature.

The injection power-dependent unpolarized, TE-polarized, and TM-polarized PL spectra are measured in the power range of 68 mW–197 mW at room temperature and displayed in Figure 3(a)–(c). Meanwhile, the injection power-dependent unpolarized, TE-polarized, and TM-polarized EL spectra are measured in the power range of 90 mW–360 mW at room temperature and exhibited in Figure 3(d)–(f). Here, the EL spectra of the nanowire–well sample were obtained by utilizing a continuous current source (LDC220C) perpendicular to the sample surface for electrical pumping. As expected, there is no obvious shift in the peak positions of the EL spectra compared to the PL spectra at almost the same injection level. The slight fluctuations in peak positions are due to the small differences between samples from different batches. Consequently, the electric-field-dependent variation in the energy band gap can be effectively mitigated in this nanowire–well structure.

**Figure 4:**
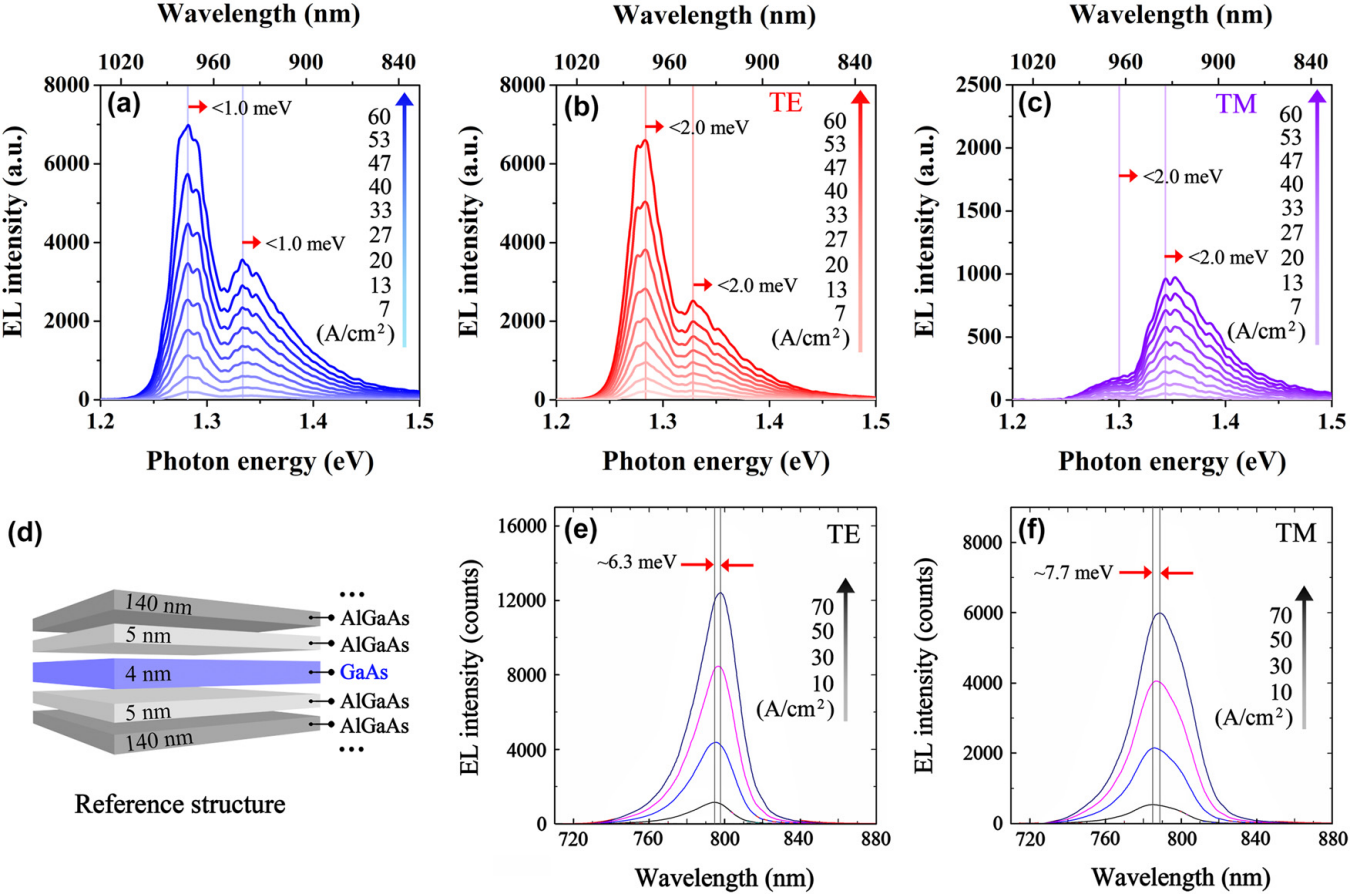
Luminescence peak characterizations of the EL spectra in the nanowire–well structure and the GaAs reference quantum well at similar injection. (a) Unpolarized, (b) TE-polarized, and (c) TM-polarized EL spectra of the nanowire–well structure at room temperature with the injection current density from 7 A/cm^2^ to 60 A/cm^2^. (d) Schematic diagram of the structure composition of the GaAs reference quantum well. (e) TE-polarized and (f) TM-polarized EL spectra of the GaAs reference quantum well at room temperature with current density increasing from 10 A/cm^2^ to 70 A/cm^2^ [45].

To further illustrate the suppression of the electric-field-dependent variation in energy band gap in the nanowire–well gradient quantum system, the unpolarized, TE-polarized, and TM-polarized EL spectra of the nanowire–well structure at room temperature with a large injection current density range from 7 A/cm^2^ to 60 A/cm^2^ are measured, as shown in Figure 4(a)–(c). In addition, a traditional GaAs quantum well, as a reference structure, has been studied for its energy band gap variation with the electric field, as displayed in Figure 4(d)–(f). For the nanowire–well gradient quantum system, there is no obvious shift in the luminescence peak of the EL spectra with the gradually increased injection. Even at high current injection, that is, under the action of a strong electric field, the structure should theoretically be affected by a strong QCSE effect, so the luminescence peak should have a very obvious shift, but it does not appear in our experiment. Specifically, the energy band gap changes of the pure-well part and the nanowire–well part are both <1.0 meV for the unpolarized EL spectra of the nanowire–well system with an injection current density range from 7 A/cm^2^ to 60 A/cm^2^. For the TE- and TM-polarized EL spectra of the nanowire–well system, the energy band gap changes are both <2.0 meV. The change rate of the energy band gap with injection current in the nanowire–well gradient system is <18.9 μeV cm^2^/A for the unpolarized property. For the TE and TM polarization modes, the sensitivity to current injection is <37.7 μeV cm^2^/A. Due to the lack of an In-segregation effect, the GaAs quantum well, as a traditional quantum system rather than a gradient quantum system, is used as a reference structure. The schematic diagram of the epitaxial kernel structure of the GaAs quantum well is shown in Figure 4(d). The active material is a 4 nm-thick unstrained GaAs single quantum well, which is sandwiched by 5 nm-thick Al_0.25_Ga_0.75_As barrier layers [45]. The TE- and TM-polarized EL spectra of the GaAs quantum well are measured under a continuous current source (LDC210C) in a current density range of 10 A/cm^2^–70 A/cm^2^ at room temperature, as shown in Figure 4(e) and (f) [45]. After measurement, within the range of current density variation, for the GaAs quantum well, the changes in band gap under TE and TM polarization modes are ∼6.3 meV and ∼7.7 meV, respectively. Correspondingly, the change rates of band gap are ∼105.0 μeV cm^2^/A and ∼128.3 μeV cm^2^/A. Here, we define the efficiency improvement in the band gap stability (*η*) of the nanowire–well gradient system compared to the GaAs quantum well as:

(1) $$\eta =\left|\frac{R_{WW}-R_{\text{GaAs}}}{R_{\text{GaAs}}}\right|\times 100\;\%$$

where *R*_ww_ and *R*_GaAs_ represent the change rates of energy band gap in the nanowire–well gradient system and GaAs quantum well, respectively. Therefore, compared with the GaAs well, a traditional quantum system, the efficiency improvement in the band gap stability of the nanowire–well gradient system is 64.1 % and 70.6 % for the TE polarization and TM polarization, respectively. Studies have shown that using a thinner active layer can further weaken the QCSE [22], [46]. The standard treatment of the QCSE in a quantum well based on time-independent perturbation theory predicts that, to leading order, the energy band gap shift in a fixed electric field strength varies as the fourth power of the quantum well thickness. Therefore, compared to the 12 nm-thick nanowire–well layer, the QCSE in the thinner GaAs quantum well layer (4 nm thick) should be more strongly suppressed. However, the QCSE has been further suppressed in the nanowire–well gradient system, and the energy band gap stability has been greatly improved compared to that of the GaAs well. By comparing the energy band gap variation of the nanowire–well sample injected with light and that injected with electricity, as well as comparing the energy band gap variation of the electrically injected nanowire–well sample and the electrically injected GaAs quantum well sample, the band gap changes induced by QCSE in nanowire–well gradient quantum systems are effectively mitigated.

In addition, as a typical GaAs/In_0.17_Ga_0.83_As/GaAs material system, the large lattice constant between these two materials will induce significant compressive stress in the pure InGaAs quantum well. In order to weaken the influence and interference of the strain-driven built-in piezoelectric polarization electric field on the QCSE and more accurately consider the mechanism of mitigating the QCSE in our specially designed gradient quantum system based on energy band engineering, the nanowire–well part in the nanowire–well structure is designed. In the nanowire–well part, due to the gradual increase of In-content along the growth direction, the lattice of the nanowires is larger than that of the quantum well, so the well is subjected to the tensile stress from the nanowires. Therefore, the tensile stress is introduced into the compressively strained quantum well, and the tensile stress counteracts part of the compressive stress in this well; thus, the influence of the stress impact in the nanowire–well part is very weak. These related explanations have been illustrated in detail in the previous work [32].

**Figure 5:**
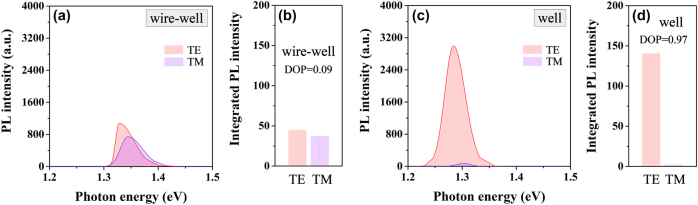
Polarization characteristics of the nanowire–well part and the pure-well part. (a) TE- and TM-polarized PL spectra of the nanowire–well part in the nanowire–well structure. (b) The integrated PL intensities of the TE- and TM-polarized PL spectra in the nanowire–well part. (c) The polarized PL spectra of the pure-well part in the nanowire–well structure. (d) The integrated PL intensities of the polarized PL spectra in the pure-well part.

To clarify this weak stress effect in the nanowire–well part, the TE- and TM-polarized PL fitting spectra of the nanowire–well part are displayed in Figure 5(a), as well as the integrated PL intensities of the TE- and TM-polarized PL spectra in this part are illustrated in Figure 5(b). Meanwhile, for comparison, the TE- and TM-polarized PL fitting spectra of the pure-well part are exhibited in Figure 5(c), as well as the integrated PL intensities of the TE- and TM-polarized PL spectra in this part are illustrated in Figure 5(d). It is very obvious that there is only a very small difference in the spectral intensity of TE and TM polarization modes in the nanowire–well part, but this difference is very large in the pure-well part. The degree of polarization (DOP), defined as DOP = (*I*_TE_ − *I*_TM_)/(*I*_TE_ + *I*_TM_) [47], [48], [49], is usually used to reflect the strain strength in the structure. Here, *I*_TE_ and *I*_TM_ denote the integrated PL intensities of TE polarization and TM polarization, respectively. With the data in Figure 5(b) and (d), the calculated DOP values are 0.09 and 0.97 for the emissions from the nanowire–well part and the pure-well part, respectively. The very low DOP indicates the comparable TE and TM intensities in the nanowire–well part, suggesting that the stress action in this part is very weak, and this part is close to a no-strain part. Therefore, the strain-driven built-in piezoelectric polarization electric field is extremely weak in this nanowire–well part. Thus, when an additional electric field is applied to this system through external devices, there is no interference from the built-in electric field. It also eliminates the possibility that the built-in electric field and the external electric field have opposite directions, resulting in the cancellation of the electric field strength acting on the active region of the sample. Therefore, temporarily, it can be considered that when the nanowire–well part serves as the investigation target, the mitigation mechanism of this gradient nanowire–well structure on the energy band gap variation induced by QCSE can be analyzed more clearly.

**Figure 6:**
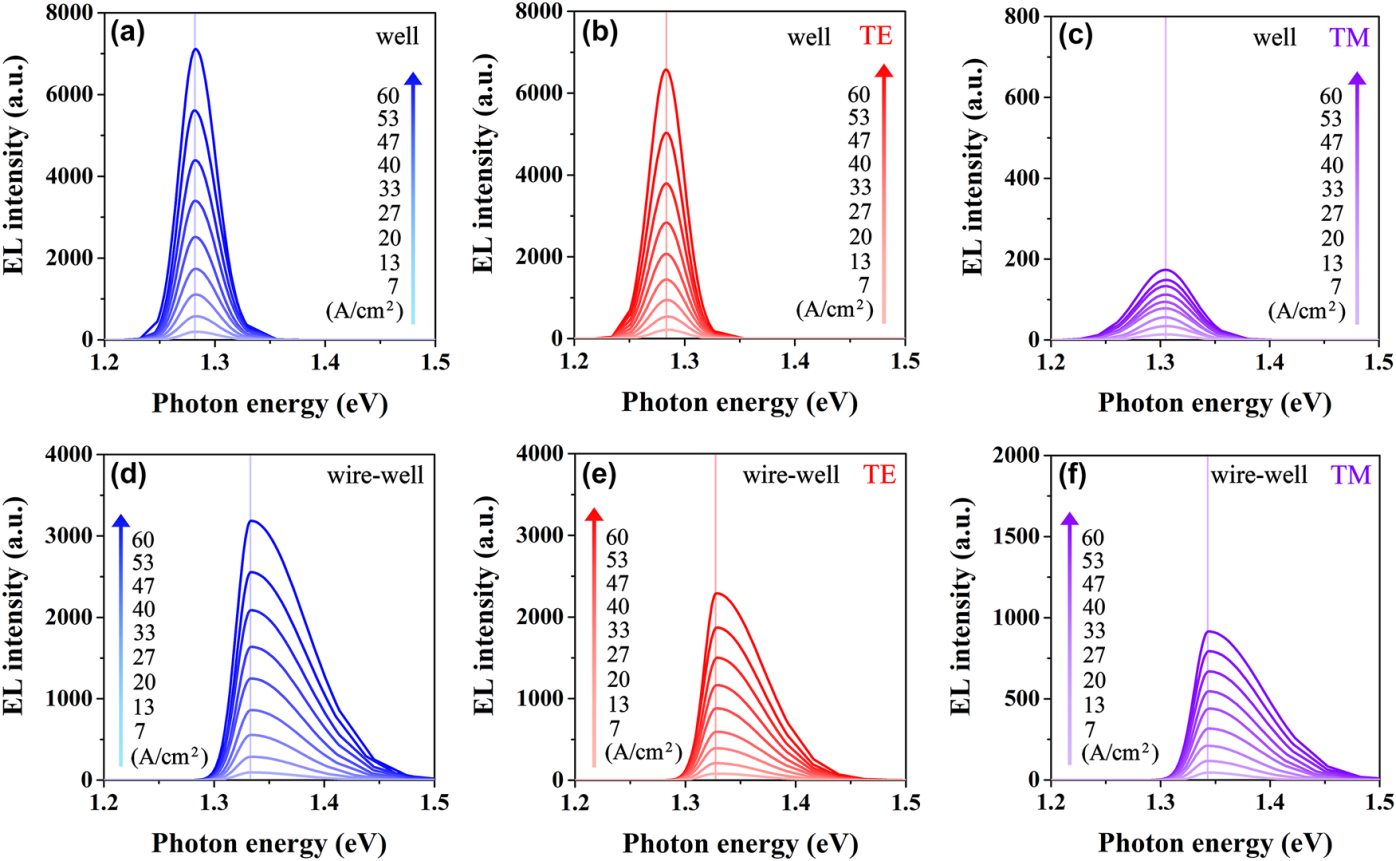
Luminescence peak characterizations of the EL fitted spectra in the pure-well part and the nanowire–well part at similar injection. (a) Unpolarized, (b) TE-polarized, and (c) TM-polarized EL fitted spectra of the pure-well part at room temperature with injection current density increasing from 7 A/cm^2^ to 60 A/cm^2^. (d) Unpolarized, (e) TE-polarized, and (f) TM-polarized EL fitted spectra of the nanowire–well part at room temperature with injection current density increasing from 7 A/cm^2^ to 60 A/cm^2^.

To distinguish the luminescence peaks of the pure-well part and the nanowire–well part from the nanowire–well sample, the EL spectra of the nanowire–well sample are fitted by bi-Gaussian fitting. The injection current density-dependent unpolarized, TE-, and TM-polarized EL fitted spectra of the pure-well part are shown in Figure 6(a)–(c), as well as the injection current density-dependent unpolarized, TE-, and TM-polarized EL fitted spectra of the nanowire–well part are depicted in Figure 6(d)–(f), respectively. There is no obvious change in the energy band gap, not only for the pure-well part with a built-in electric field but also for the nanowire–well part without a built-in electric field.

The electric-field-independent energy band gap is obtained in both the nanowire–well part and the pure-well part, which indicates that the mechanism of mitigating the variation of energy band gap with electric field induced by the QCSE can target both strain-driven built-in piezoelectric polarization electric fields as well as external electric fields under electrical injection. Utilizing the In-segregation effect, based on the energy band engineering, the electric-field-independent energy band gap is obtained in this InGaAs nanowire–well structure, which provides a new idea for the realization of frequency-stable laser devices and other high-performance optoelectronic devices.

### 3.2 Mechanism of mitigating electric-field-dependent energy band gap variation

**Figure 7:**
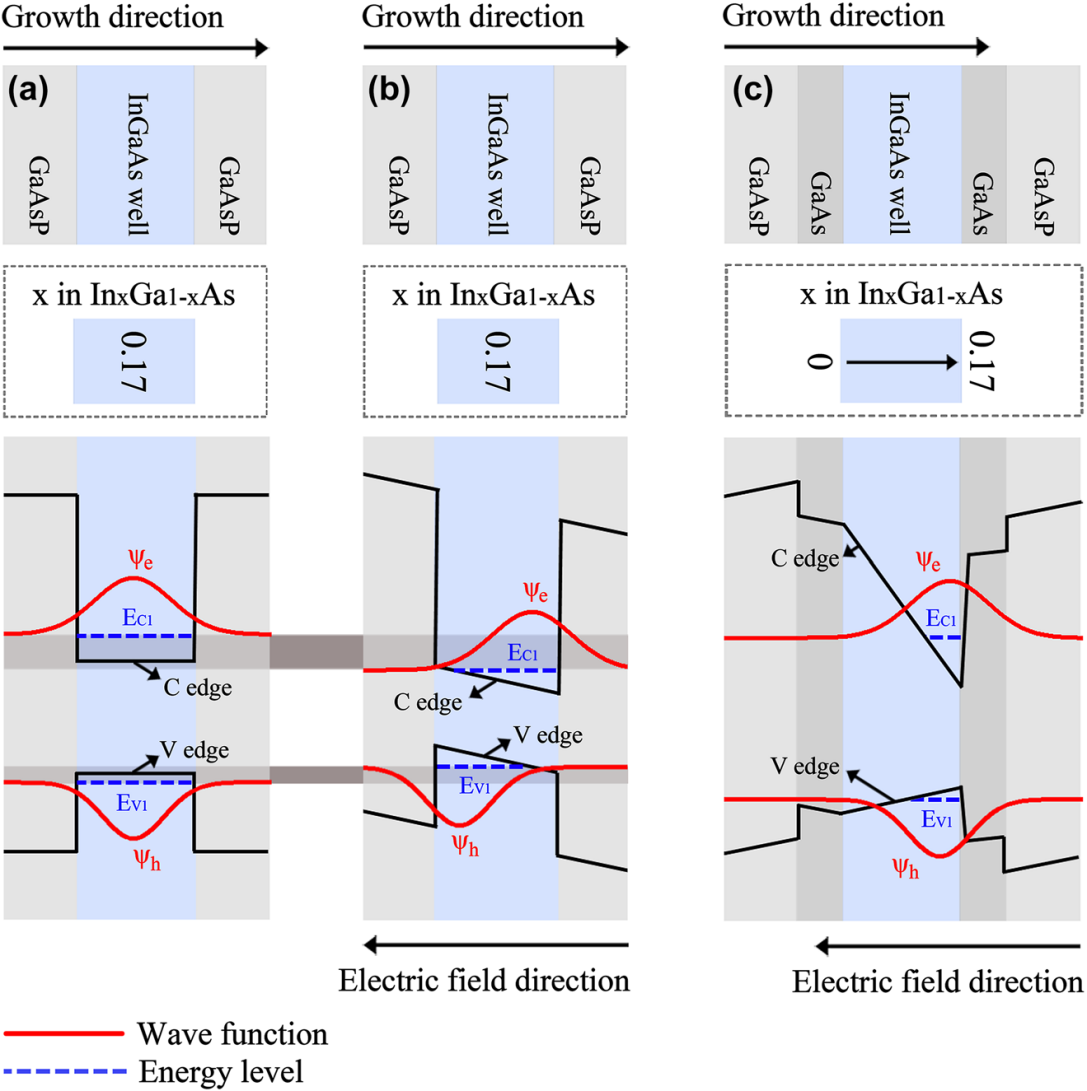
Mechanism of mitigating electric-field-dependent energy band gap variation. Schematic representation of the kernel epitaxial structure, In-content in InGaAs active layer, and energy band structure in (a) an ideal InGaAs quantum well without electric field and stress impacts, (b) a strained InGaAs quantum well with an external applied electric field, and (c) the pure-well part in the nanowire–well structure with an external applied electric field.

To illustrate the mitigation mechanism of the nanowire–well structure on the electric-field-dependent energy band gap variation induced by the QCSE, the energy band together with electron and hole wave function diagrams of an ideal In_0.17_Ga_0.83_As quantum well without electric field and stress impacts as well as a strained In_0.17_Ga_0.83_As quantum well with a vertical external applied electric field are drawn and presented in Figure 7(a) and (b), respectively. The energy band edges in the unstrained well of Figure 7(a) are flat due to the absence of the electric field impact, but in the quantum well with the external applied electric field and stress impacts, as exhibited in Figure 7(b), the conduction band (denoted as *C* in the figure) and valence band (denoted as *V* in the figure) edges are inclined and tilt in the same direction. This tilt of band edges induced by electric fields makes the energy band gap of the active material decrease compared to the ideal well without the electric field impact in Figure 7(a).

To mitigate electric-field-dependent energy band gap variation induced by the QCSE, we propose a gradient quantum system that features a design of continuously increased In-content along the material growth direction based on the In-segregation effect. Here, the pure-well part of the nanowire–well structure is selected as an example for the following analyses. The energy band structure diagram of the pure-well part is illustrated in Figure 7(c). It could be found that the conduction (*C*) and valence (*V*) band edges in this system tilt in different directions due to the continuously increased In-content along the material growth direction. Furthermore, for the valence band in this system, the band edge tilt caused by the In-segregation effect and the band edge tilt caused by the electric field are in two opposite directions, so this special design of gradient In-content can compensate for the valence band tilt caused by the electric field to some extent. Therefore, this modified energy band profile in the gradient quantum system makes the energy band gap of the active material almost invariant with the change in the electric field. So the electric-field-dependent energy band gap variation can be effectively mitigated, which provides a feasible way to develop frequency-stable laser diodes and increase their practical value.

## 4 Conclusions

In summary, a method for mitigating the electric-field-dependent energy band gap variation induced by the QCSE based on the nanowire–well structure with a gradient In-content is introduced in this paper. In this special structure, the energy band profile is modified by the In-segregation effect, which effectively mitigates the variation of the energy band gap with the electric field to some extent. Experimental results indicate that the energy band gap of the active material remains relatively stable at the identical injection level in both electric injection samples that are subjected to the vertical electric field and in the optical injection samples without the electric field impact. Meanwhile, as the external electric field is gradually increased, there is also no obvious change in the energy band gap in the electric injection samples. Importantly, compared with a thinner GaAs reference quantum well that theoretically has a stronger weakening effect on the QCSE, the nanowire–well gradient system exhibits stronger band gap stability and a higher band gap stability efficiency. This realization of electric-field-independent energy band gap in the InGaAs nanowire–well gradient system brings a new opportunity for the realization of frequency-stable laser diodes and enhances the application value of quantum-confined optoelectronic devices.
